# Artificial Neural Networks Based Controller for Glucose Monitoring during Clamp Test

**DOI:** 10.1371/journal.pone.0044587

**Published:** 2012-08-31

**Authors:** Merav Catalogna, Eyal Cohen, Sigal Fishman, Zamir Halpern, Uri Nevo, Eshel Ben-Jacob

**Affiliations:** 1 Department of Biomedical Engineering, Faculty of Engineering, Tel-Aviv University, Tel-Aviv, Israel; 2 HEMDA, Center of Science Education, Tel-Aviv, Isreal; 3 Department of Gastroenterology and Hepatology, Tel Aviv Sourasky Medical Center, Tel Aviv, Israel, Affiliated to Sackler School of Medicine, Tel Aviv, Israel; 4 School of Physics and Astronomy, Tel-Aviv University, Tel-Aviv, Israel; Technical University of Madrid, Italy

## Abstract

Insulin resistance (IR) is one of the most widespread health problems in modern times. The gold standard for quantification of IR is the hyperinsulinemic-euglycemic glucose clamp technique. During the test, a regulated glucose infusion is delivered intravenously to maintain a constant blood glucose concentration. Current control algorithms for regulating this glucose infusion are based on feedback control. These models require frequent sampling of blood, and can only partly capture the complexity associated with regulation of glucose. Here we present an improved clamp control algorithm which is motivated by the stochastic nature of glucose kinetics, while using the minimal need in blood samples required for evaluation of IR. A glucose pump control algorithm, based on artificial neural networks model was developed. The system was trained with a data base collected from 62 rat model experiments, using a back-propagation Levenberg-Marquardt optimization. Genetic algorithm was used to optimize network topology and learning features. The predictive value of the proposed algorithm during the temporal period of interest was significantly improved relative to a feedback control applied at an equivalent low sampling interval. Robustness to noise analysis demonstrates the applicability of the algorithm in realistic situations.

## Introduction

Insulin resistance syndrome (IRS) is one of the most widespread health problems in modern times, and is the main cause of type II diabetes. The clinical manifestations of the syndrome include abnormal plasma insulin levels, hypertension, dyslipidemia and glucose intolerance. IRS is the main cause of type II diabetes and can also progress to obesity, cardiovascular disorders, non-alcoholic fatty liver disease, and polycystic ovary syndrome. Lifestyle habits, chronic use of certain medications [1] as well as genetic factors are assumed to be some of the pivotal causes of insulin resistance (IR) and type II diabetes. Since no known and proven cure currently exists, treatment focuses on controlling the symptoms: regulation of blood glucose levels, control of weight, and maintenance of healthy blood fat levels [2]. In 2007 the United Nations has officially recognized diabetes as a global epidemic which requires allocation of resources for prevention and treatment. According to the World Health Organization (WHO) estimation, more than 220 million people worldwide and around 20 million people in the US alone suffer from diabetes. A worldwide dramatic rise is predicted, with a forecast growth rate of almost 40% in twenty years [3], [4]. Due to the growing interest in treatment and prevention of IRS and type II diabetes, quantification of insulin resistance is critical for both clinical and research proposes.

The gold standard method for quantifying insulin resistance is the hyperinsulinemic-euglycemic glucose clamp (HEGC) technique. In this method, plasma glucose and plasma insulin concentrations are controlled by the investigator and thus the natural glucose-insulin feedback loop is interrupted and directed. During the test, plasma insulin is raised acutely to a desired set-point and maintained at that level throughout the study due to constant exogenous insulin infusion. In response to insulin action, glucose infusion is administrated in order to maintain (‘clamp’) plasma glucose within its euglycemic (fasting) level. Whole-body insulin resistance, can be calculated, under of the approximation of steady-state conditions of glucose and insulin levels. Thus, the exogenous glucose infusing rate (GIR) can serve as an estimation of the net glucose disposal rate (Rd) [5]. In order to maintain plasma glucose at the desired level, the HEGC test is performed such that the investigator manually sets the glucose infusion rate. To improve accuracy of this feedback loop, several real time computer-based algorithms have been developed for controlling glucose infusion rate in response to frequently measured plasma glucose levels.

DeFronzo *et al.* [5] proposed a negative feedback algorithm for real time calculation of glucose infusion rate based on volume and metabolic modifications in glucose concentration. Later, Clemens *et al.* have introduced the Biostator Glucose Controlled Insulin Infusion System (GCIIS) [6]. The automatic system features a closed-loop glucose algorithm. It provides glucose and insulin pumps control, and blood glucose concentration monitoring sensor with one minute sampling rate. Another simple solution was the algorithm proposed by Furler *et al.* [7]. The algorithm employs proportional and differential feedback control and uses discrete blood sampling with a sampling rate of five minutes interval. Lately, Bequette [8] has suggested a simple model-based glucose proportional integral controller with time-delay compensation.

Glucose metabolism is affected by many factors such as peripheral and hepatic sensitivity, physical activity level, diet, stress and endogenous control systems. Current mathematical models cannot fully describe the complex physiological regulation and the subject's metabolic state. Furthermore, use of such models for maintaining a desired glucose level in a HEGC test requires frequent sampling of blood glucose. In small sized animals, blood volume is limited and it is necessary to reduce the withdrawal of blood samples to minimum in order to prevent intravascular volume depletion and stress.

**Table 1:** Data Groups Characteristics.

Data Set	Rat Species	n	BW [g]	BPG [mg/dL]
Training	Wistar HAN	82	301±2	133.9±1.6
	SD	20	319±4	127.5±1.1
	Fischer-F344	81	255±3	129.2±1.8
Validation	Wistar HAN	28	298±4	131.9±3.3
	SD	5	323±4	127.7±3.2
	Fischer-F344	28	249±5	130.2±3.3
Test	Wistar HAN	27	292±3	133.8±4.1
	SD	5	322±9	123.0±4.2
	Fischer-F344	29	251±5	130.6±3.1

SD - Sprague Dawley, BW - body weight, BPG - average basal plasma glucose concentration.

**Figure 1:**
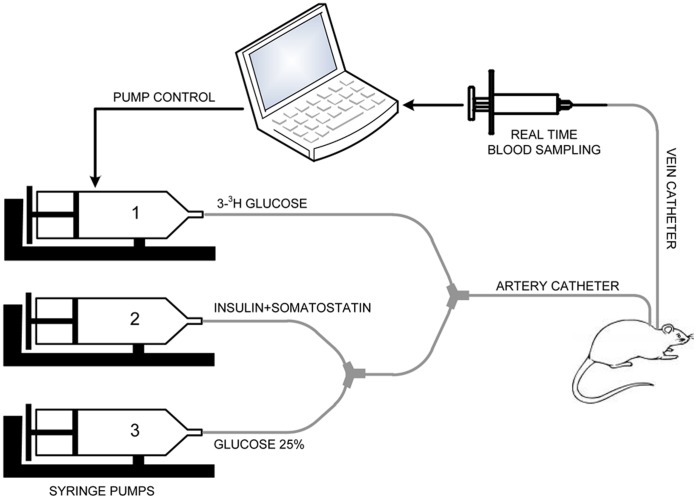
Schematic configuration of the experimental setup. The system consists of three infusion syringe pumps for [3-^3^H] glucose, insulin and variable glucose respectively. Arterial catheter is connected to the infusion pumps, and venous catheter is used for manual blood sampling. A closed-loop, computer controlled system is proposed for maintaining plasma glucose concentration within the desired level during HEGC.

In the present work, we aim at developing an algorithm for real time automatic regulation of glucose levels during HEGC, by taking into account the stochastic nature of glucose kinetics and the nonlinear relationship between input parameters [9]. In addition, the algorithm should be performed with the use of minimum blood samples required for evaluation of IR. An artificial neural networks (ANN) algorithm was suggested to develop a predicting controller for regulating glucose infusion rate as part of a closed-loop system. The controller was trained and evaluated on experimental data collected during HEGC tests in rat models. This database included results obtained from previously reported experiments [10], [11], as well as results collected in experiments we have conducted and are reported here for the first time.

**Figure 2:**
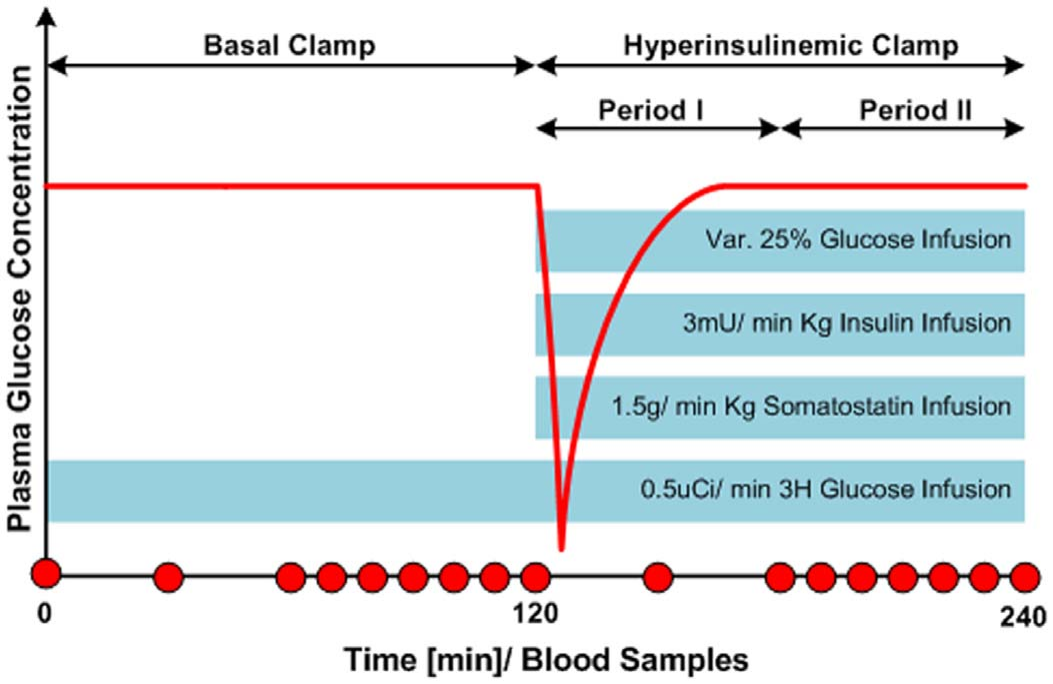
Hyperinsulinemic-euglycemic glucose clamp experiment (HEGC) protocol – Schematic illustration. Representation of the experimental design of the clamp study: The animals were studied under basal conditions for the first 2 hours and under hyperinsulinemic conditions over the last 2 hours. Period–I is characterized by rapid changes in glucose concentration. Period-II exhibits a near steady state behavior of insulin and plasma glucose concentrations. Circles represent times at which blood samples were taken.

**Table 2:** Network Parameters and Ranges.

Parameter	Lower Bound	Upper Bound
N_L_	1	3
[N_CELL_]	2	25
μ_0_	10^−4^	2
μ^−^	0.01	0.95
μ^+^	1.05	10

N_L_ - number of layers, [N_CELL_] - number of cells in each layer vector, μ_0_, μ^+^, μ^−^ - Levenberg-Marquardt optimization learning parameters.

## Methods

### Ethics Statement

The animals were treated and handled according to the guidelines of Tel-Aviv Sourasky Medical Center’s Animal Care and Use Committee. The study protocol was reviewed approved by the Animal Care and Use Committee of the Tel Aviv Sourasky Medical Center, permit number: 27_09_08.

### Animal Model

In order to obtain a wide range of insulin sensitivity levels, the datasets of this study were collected from 62 experiments performed using a similar HEGC protocol. Table 1 lists the characteristics of the animals. A part of the experimental results was obtained from pervious data sets using Sprague Dawley and Fischer-F344 rats (recorded by colleagues from the Dept. of Gastroenterology and Hepatology, TASMC, as detailed in [10], [11]). Another part of the experiments was performed by the authors using Wistar Han rats (Harlan, Jerusalem). In our experiments, variations in insulin sensitivity levels were achieved by using short duration peripheral electrical stimulation (PES) applied non-invasively at functional skin zones at the hind-limbs (Frequency 2Hz/bursts 16 Hz rectangular bi-phasic, Pulse width: 150 µs, 5–10 mA variable). Detailed information about this study is in preparation for publication.

**Figure 3:**
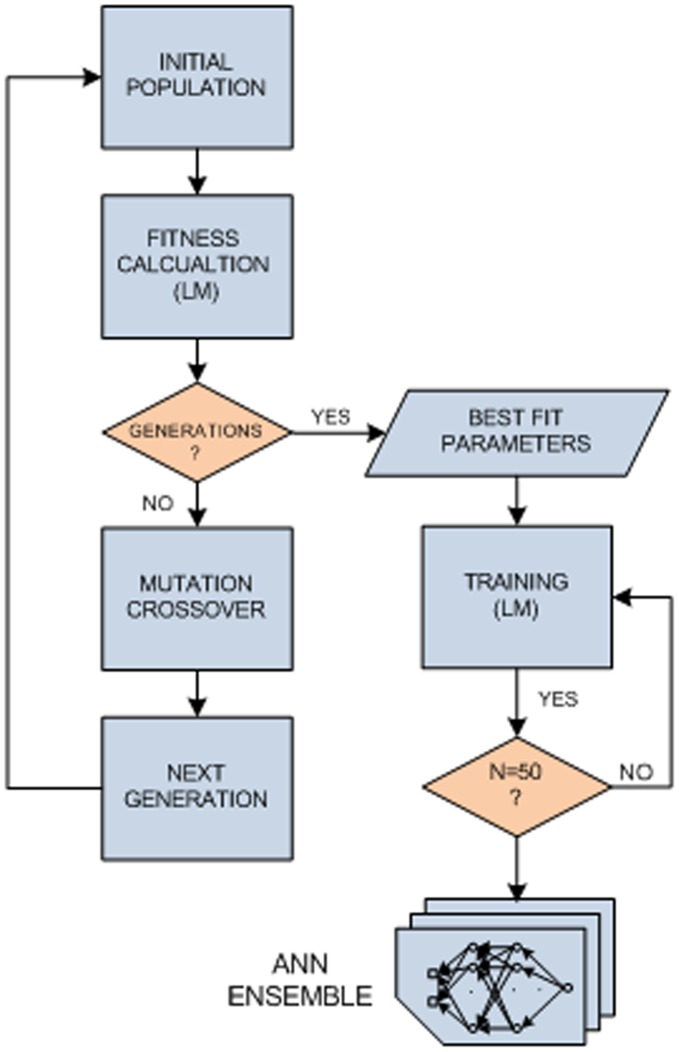
Glucose pump controller design stage block diagram. The output of this stage is an ensemble of 50 sets of Artificial Neural Network (ANN) connection weights, created using the Test set of data and the best fit parameters vector.

**Table 3:** Animals Characteristics.

Rat Species	Wistar HAN	SD	Fischer-F344
N	28	6	28
BW [gr]	299±4	320±7	252±6
BPG [mg/dL]	133.4±3.2	128.6±2.8	131.0±3.2
GIR [µl/min]	5–22	10–21	5–15
Total No of vectors: Period-I	55	12	52
Total No of vectors: Period-II	137	30	138

SD - Sprague Dawley, BW - body weight, BPG - average basal plasma glucose concentration, GIR - glucose infusion rate.

#### Hyperinsulinemic-euglycemic clamp (HEGC) protocol

All studies were performed as described in previous studies [12], [13]. In brief, the studies were performed in conscious, unstressed and unrestrained rats using HEGC technique, in combination with high-performance liquid chromatography–purified [3-^3^H] glucose infusion. The experimental setup of the system is illustrated in Figure 1. The system consists of three infusion syringe pumps (NE-1000, Syringe Pump Inc) for [3-^3^H] glucose, insulin and variable glucose respectively. Arterial catheter is connected to the infusion pumps, and venous catheter is used for manual blood sampling.

The studies lasted 240 minutes and included two hours of basal clamp phase for assessment of the basal glucose turnover, and two hours of hyperinsulinemic-euglycemic clamp phase for evaluation of insulin sensitivity. At the beginning of the basal clamp phase, a primed-continuous infusion of [3-^3^H] glucose (10 µCi bolus, 0.5 µCi/min; Perkin-Elmer, Boston, MA) was initiated and maintained throughout the remaining 4 hours of the study. At the beginning of the hyperinsulinemic phase, a primed-continuous infusion of insulin (3 mU/min Kg) was administered, and a variable infusion of a 25% glucose solution was started and later on adjusted manually and periodically to clamp the plasma glucose concentration at the basal level. To prevent endogenous insulin secretion, somatostatin (1.5 g/min Kg) was infused. Plasma samples for glucose measurements were obtained at t = 0, 30, 60, 70, 80, 90, 100, 110 and 120 minutes during the basal phase and at t = 150, 180, 190, 200, 210, 220, 230, and 240 minutes during the glucose clamp phase. A schematic representation of the study protocol is shown in Figure 2. Serum glucose concentration was determined using ELISA (BioVision Research Products, Mountain View, CA).

**Figure 4:**
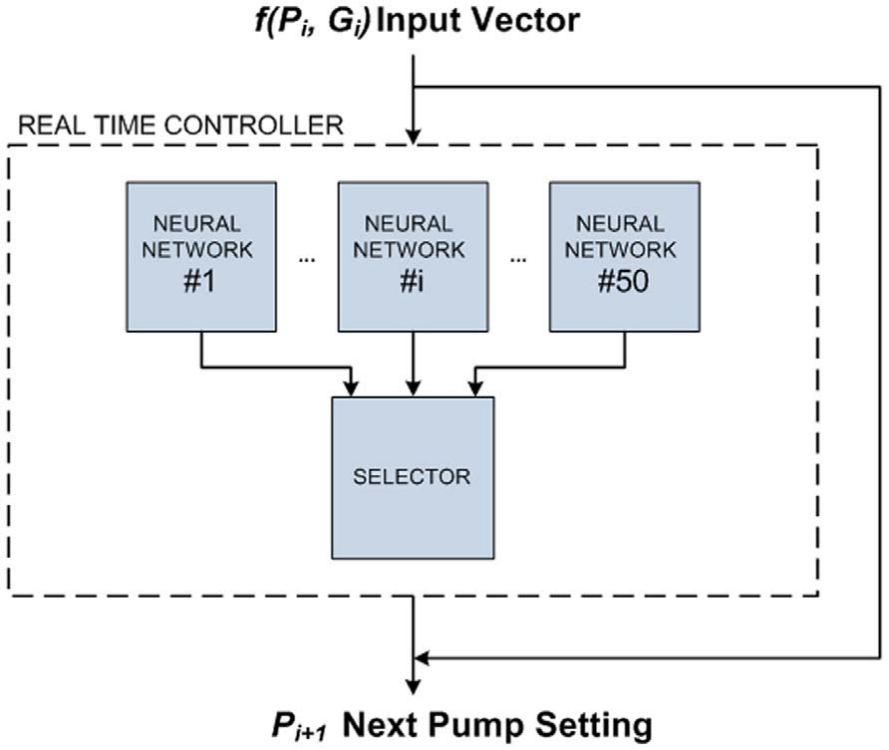
Real time glucose pump controller block diagram. In each time slot in a real time experiment, an input vector *f(P_i_,G_i_)* is calculated where *P_i_* is the pump setting and *G_i_* is the blood glucose level of step i. The controller’s prediction output *P_i+1_* is derived as the median of 50 predictions.

**Table 4:** Optimized Parameters and Best Performance.

Parameter	Period-I	Period-II
Architecture	7–4–1	8–2–5–1
μ_0_	0.5	1.5
μ^+^	0.82	0.85
μ^−^	1.55	5.9
RMSE	1.383	0.597
cc	0.9150	0.9904
% Error	8.75±0.97	3.69±0.47

μ_0_, μ^+^, μ^−^ - Levenberg-Marquardt optimization learning parameters, RMSE - root mean square error, cc - correlation coefficient.

### Neural Networks Model Design

#### Data acquisition

The data set, collected for each animal, consisted of the following information: rat's body weight, 16 plasma glucose concentration levels (8 during the basal clamp phase and 8 during the hyperinsulinemic-euglycemic clamp phase, both in fixed time slots) and 8 glucose pump settings corresponding to each time slot of measurement during the hyperinsulinemic euglycemic-clamp phase.

**Figure 5:**
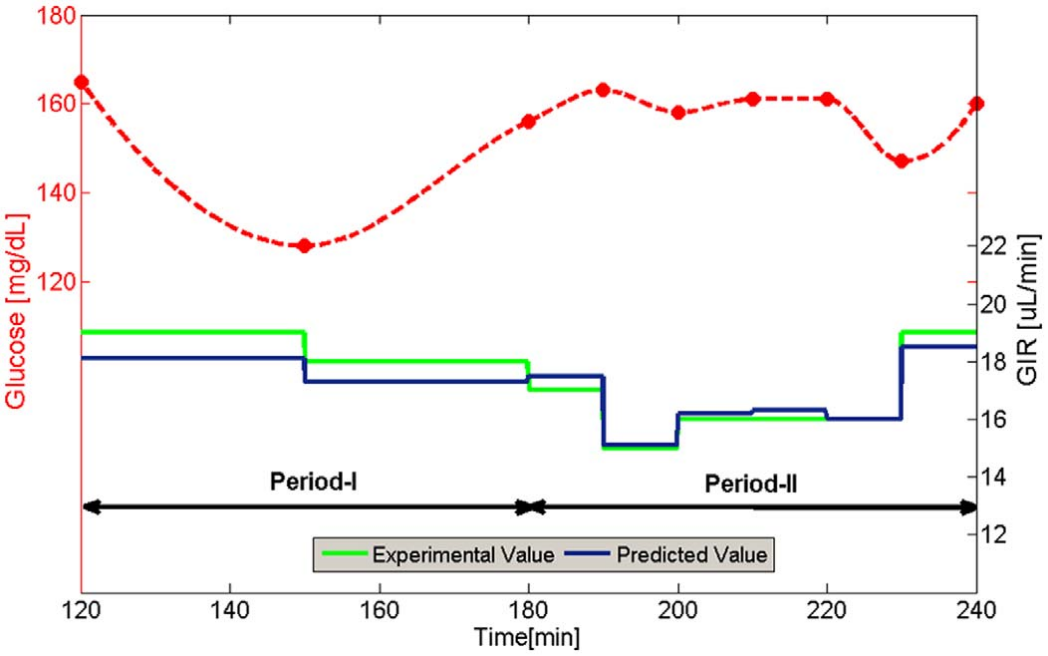
Artificial neural networks model predictive performance. Plasma glucose concentration and experimental glucose infusion rate during hyperinsulinemic-euglycemic glucose clamp phase in comparison to target values (data presented for animal TG11).

**Figure 6:**
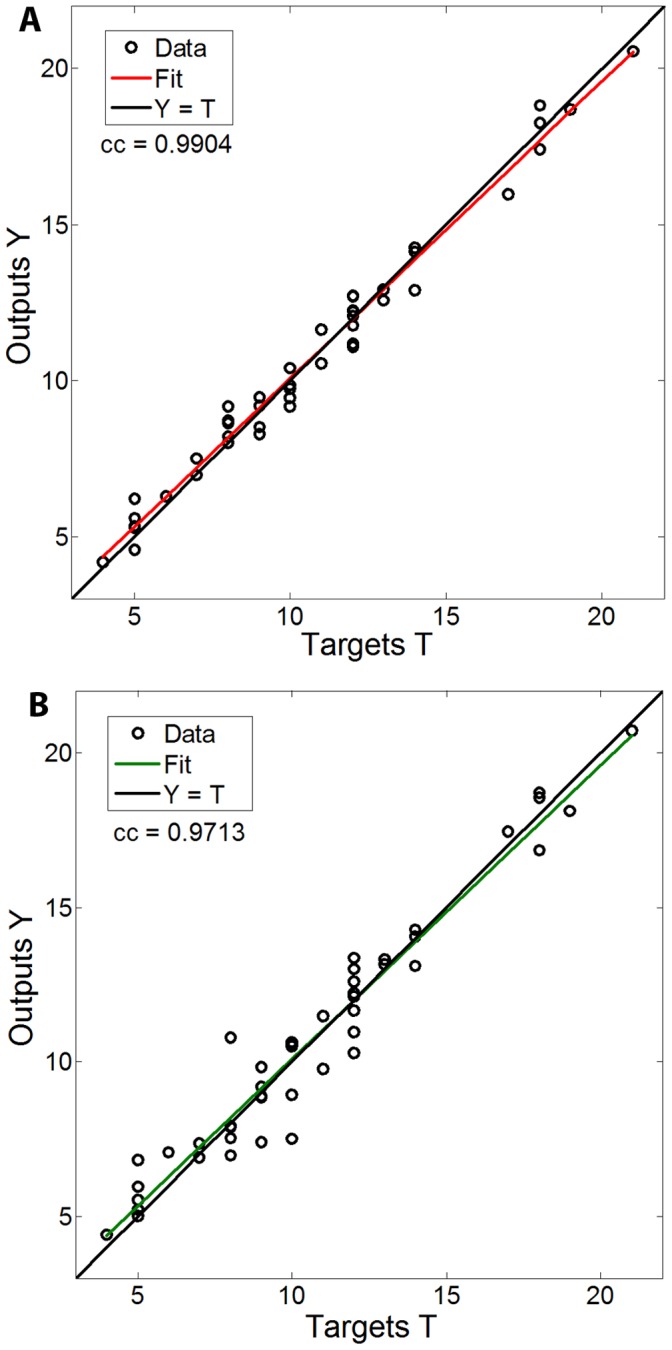
Regression analysis between the predicted and desired values of the ANN glucose pump controller. Performance results of the Test set simulation. **A**: ANN trained using Levenberg-Marquardt (LM) optimization algorithm, **B**: ANN trained using Gradient-Descent with momentum and adaptive learning rate algorithm.

**Figure 7:**
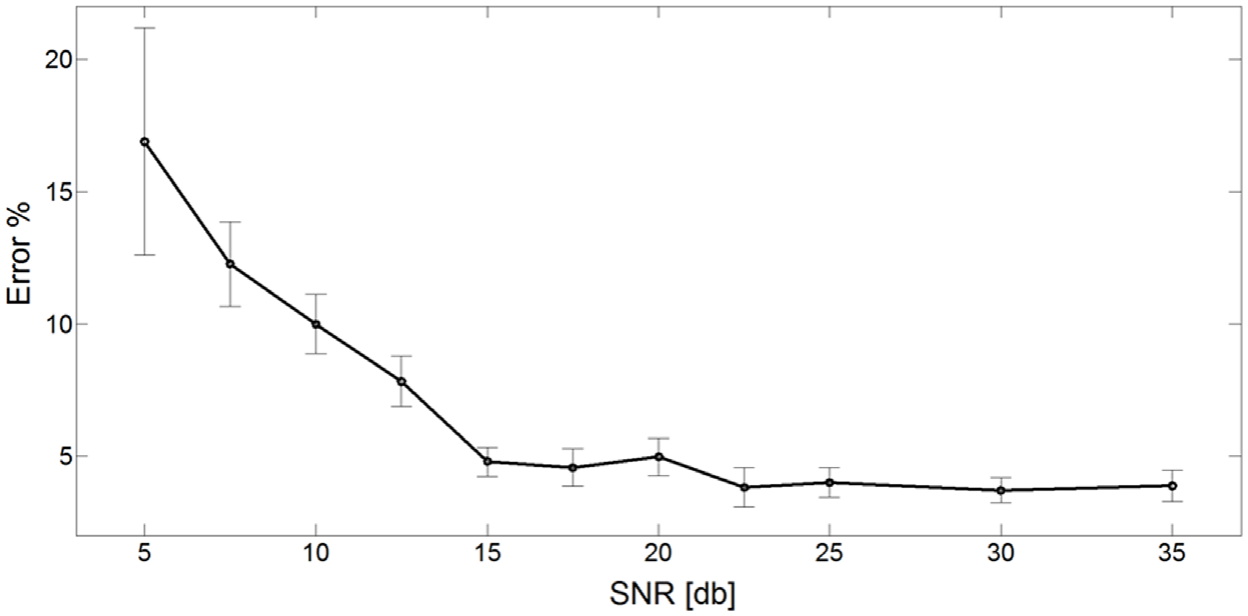
Evaluation of the error in the prediction of glucose infusion rate over different levels of random noise. Random Gaussian density function noise with zero mean, and variance corresponding to signal to noise ratios (SNR) of 5 dB to 35 dB was added to the input data. The prediction error is expressed in mean ± SEM over 100 simulations.

**Figure 8:**
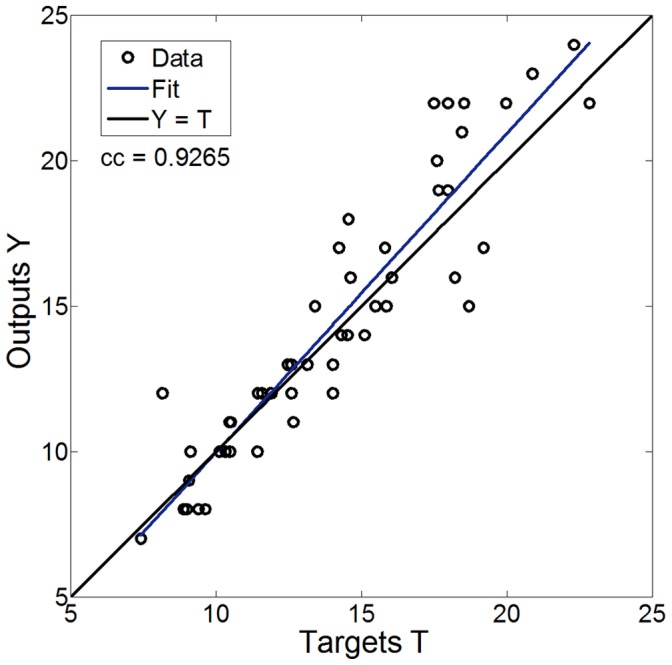
Regression analysis between the predicted and desired values calculated using feedback control algorithm. The feedback control algorithm of DeFronzo *et al*. [5] was used for performance comparison, with a sampling rate of 10 minutes interval.

#### Input data vectors

In order to realize the glucose pump controller algorithm during the hyperinsulinemic-euglycemic clamp phase, two neural networks were suggested representing two states of physiological behavior (Figure 2). During the first period (Period-I), plasma insulin concentration is raised acutely to a new level while a variable glucose infusion keeps the blood glucose concentration in its euglycemic level. Period-I is characterized by rapid changes in glucose concentration in order to stabilize the glucose levels on the basal concentration. The second period (Period-II) starts at T = 190 minutes from initiation of the study, and exhibits a near steady state behavior of insulin and plasma glucose concentrations. The input data and the output vectors are given by
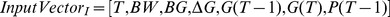
(1)

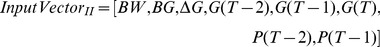
(2)

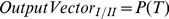
(3)where *T* is the time slot in minutes, *BW* is the rat body weight, *BG* is the mean basal plasma glucose level (*PG*), calculated over the last hour of the basal clamp phase, Δ*G* is the difference between the current *PG* level and the desired *PG* level, *G(T)* is the plasma glucose at time slot *T* and *P(T)* is the glucose pump setting at time slot *T*.

For each period, the chosen parameters of the training vector were uncorrelated using principal component analysis (PCA) technique, and contributed more than 0.01% of the total variance in the database. Data was normalized by scaling the values within the range [−1 1] for each parameter. The data vectors were randomly assigned into three groups – Training, Validation and Test sets. Table 2 lists the general characteristics of the groups.

#### Learning strategy

We implemented a feed-forward multilayer artificial neural network with a back-propagation training method. Three different optimization training algorithms were tested: The Gradient-Descent with momentum and adaptive learning rate, the Scaled Conjugate Gradient and the Levenberg-Marquardt (LM) optimization algorithm. The LM algorithm provided the best performance, and also has been shown to be fast and highly efficient when training small and medium size networks, especially when high precision is required [14]. The LM method combines the Gauss-Newton method, which converges quickly near the minimum, and the gradient descent method, which converges in all space but relatively slow. The weights update rule is a linear combination of both methods:
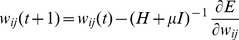
(4)where *w_ij_* are the weights of iteration *t*, *E* is the error function, *H* is the Hessian matrix of second derivatives, *I* is the identity matrix and *µ* is a positive control scalar. The algorithm starts with a large value of µ, and adjusts this value dynamically in order to decrease the error. In later iterations, as a minimum is approached, µ approaches zero and so the algorithm switches to Newton's method. The algorithm uses three parameters for process management: μ_0_ is the initial value of the control scalar, μ^+^ is a multiplier that increases the current value of μ, and μ^−^ is a multiplier that decreases the current value of μ.

The error function represents the prediction error i.e., the variation between the network’s output and the target values in the current iteration of the optimization process. The error function is based on the root mean square error (RMSE) criterion as expressed in (5).
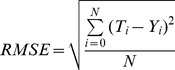
(5)where *T_i_* is the measured glucose pump setting (the Target values), *Y_i_* is the predicted value (the Output values), *N* is the total number of data records. The transfer function used is the hyperbolic tangent sigmoid function.

#### Architecture

A network’s architecture plays a key role in the capability of the system to present a desired and generalized response to a wide set of metabolic states. This is since this capability is highly responsive to the number of neurons in the hidden layers. The use of a small number of neurons may lead to under-fitting. On the other hand, too many neurons may contribute to over-fitting, in which all training vectors are well fitted, thereby, losing the generalization capability of the network and its prediction ability [15]. Therefore, the design of our glucose pump controller consisted of two stages. In the first stage we determined the best network topology and learning parameters (the ‘solution vector’), based on performance comparison measures using genetic algorithm (GA) optimization method. In the next stage an ensemble of 50 ANNs was created using the best parameters evaluated. Figure 3 illustrates the controller design algorithm.

Genetic algorithm is an iterative stochastic optimization method, and is a one type of an evolutionary algorithm. In brief, evolution process is based on a natural selection mechanism, operating over chromosomes during the reproduction stage. Across successive generations, an enhancement process is done towards creating the optimal population representing an optimal solution [16]. Initial population of potential solution vectors was generated randomly with a uniform distribution, in which each individual is represented as follows:
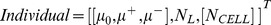
(6)where *μ_0_* is an initial value of LM learning control constant, *μ^+^* and μ^−^ are positive values that multiply the current *μ_0_* whenever the performance function was reduced or increased, respectively, in a previous step, *N_L_* is the number of hidden layers and [N_CELL_] is the number of cell elements in each layer vector. Table 3 lists the network parameters and ranges to be optimized using the genetic algorithm. According to the universal approximation theorem, ANN with one hidden layer can approximate almost any function [17]. In some cases, depending on the degree of complexity of the data, higher performance may be achieved by using larger architectures. We, therefore, examined the performance of the network in the range of 1 to 3 hidden layers. Selection of other parameters was based on previous work to avoid over-fitting. Comparison between individuals was performed through LM optimization prediction error. The next generations were created according to the following rules. We chose to use Matlab® default parameters for creating the next generations, which we found to produce optimal convergence of the algorithm.

- Best fit rule – The following generation will contain 10% of the individuals in the current population with the best fitness score.
- Crossover rule – The crossover rule is applied to 80% of the remaining individuals by combining the vectors of a pair of parents according to a random binary vector.
- Mutation rule - The mutation rule is applied to 20% of the remaining individuals by adding a random number derived from a normal Gaussian distribution to the parent vectors.

Using the optimal set of parameters obtained by the GA algorithm, an ensemble of 50 networks was generated from 50 successive LM optimization training simulations. In real time experiment for any input vector, the controller output *P_i+1_* will be chosen as the median of the ensemble output based on the performance determined by root mean square error (RMSE) and correlation coefficient (cc) values (Figure 4). The algorithm was developed using Matlab® version 7.1.

## Results

In this study, we employed artificial neural networks to develop a real time controller for automatic regulation of glucose levels during hyperinsulinemic-euglycemic glucose clamp (HEGC) test. In order to maximize the generalization performance of the controller, the proposed strategy consisted of the following components: (1) a wide range database generated from multiple sources and conditions, (2) direct determination of model architecture and learning parameters through optimization approach, and (3) stabilization element by using an ensemble networks technique. Two types of performance tests were carried out using a Test set of biological data (that was not used during the training stage). In the first test we analyzed performance using regression analysis between the ANN controller's output and the experimental target rates. In the second test we demonstrated the applicability of the algorithm in realistic situations using robustness to noise analysis. Finally, in order to evaluate the proposed method, we compared the performance of the ANN controller with a reference feedback control algorithm. The following section describes the results of our study and performance analysis.

### 

#### Animal data

The database of this study was obtained from 62 HEGC experiments. 28 experiments were performed by the authors in this study on Wistar Han rats. 34 experimental results were taken from pervious datasets of Sprague Dawley and Fischer-F344 rats as reported in [10], [11]. General characteristics of the dataset are shown in Table 3. The dataset covers a wide range of glucose infusion rate (GIR) (between 5–22 µl/min) as well as body weight and environmental conditions. A total of 120 and 300 input data vectors were generated for training Period-I and II networks respectively. Each input vector consisted of two types of variables: constant variables (body weight and basal blood glucose level) and time dependent variables (glucose pump setting and plasma glucose level at previous time slots).

#### Controller model architecture

Our proposed controller model is composed of two separate sub-units corresponding to the two states of physiological behavior during the HEGC test (Period-I, II). Each sub-unit is build of an ensemble of 50 neural networks, generated from successive training simulations. Table 4 summarizes the optimized architecture and learning parameters obtained for those neural networks by the genetic algorithm based on RMSE test function. The optimal network architecture for Period-I was composed of 1 hidden layer with 4 neurons. For Period-II, the optimal network architecture included 2 hidden layers, 2 neurons in the first layer and 5 neurons in the second layer. The ANN controller output, at any of the experimental time periods, was determined as the median of all prediction rates of the networks ensemble. For example, the variations in RMSE values, within the networks ensemble generated for the data of the Test set, were within the range 0.52 to 0.72, with an average value of 0.58±0.04. The RMSE of 60% of the networks was between 0.55 and 0.57; therefore a median value may better represent the controller’s output, and improve the system’s generalization and stability.

#### Performance analysis

Performance analysis, determined by RMSE and cc measures, was performed using the Test set. Table 4 shows performance results of the ANN controller. For Period-I ANN, the overall RMSE (calculated for the median predicted pump setting rate) and cc measures were 1.383 and 0.9150 respectively and 0.597 and 0.9904 respectively for Period-II ANN. The estimated error between the predicted pump setting value and the experimental value was 8.75±0.97% for Period-I ANN and 3.69±0.47% for Period-II ANN. As expected, better performance rates were obtained for Period-II, due to its larger training dataset. Underestimation of extreme values in the Test set led to the increased error in Period-I. However, the performance is highly acceptable for practical use, and may be improved by using larger datasets. Figure 5 demonstrates an example of the controller's prediction power, during hyperinsulinemic-euglycemic glucose clamp phase (with experimental data of a single animal, whose data was not included in the training set of data; animal number TG11). As can be seen, the pump setting rates predicted by the ANN are in good correlation with the target experimental results. This behavior is also demonstrated in a regression analysis between the ANN predicted rates and the target rates of the Test set (R^2^ = 0.9904) as shown in Figure 6. High prediction accuracy and good performance was obtained throughout the whole range of the glucose infusion rate, which represents a corresponding range of insulin sensitivity levels.

#### Robustness to noise analysis

In order to assess the behavior of the algorithm in a realistic situation, we tested the performance related to the robustness to noise in the data. We added random noise components to the Test set input vectors, drawn from a Gaussian density function with zero mean and variance corresponding to signal to noise ratios (SNR) of 5 dB to 35 dB. The average error, over 100 simulations, between the prediction rates of the ANN controller and the experimental target rates are shown in Figure 7 as a function of SNR. It appears that under severe noise conditions (SNR = 10 dB) the error is less than 10%. This property of the system of robustness to noise, further demonstrates the applicability of the algorithm with noisy biological data.

#### Performance comparison

For comparison with a reference feedback control algorithm, we implemented the algorithm by DeFronzo *et al.* [5] that was calculated with a similar blood sampling rate of 10 minutes. This algorithm for periodic real-time adjustment of glucose infusion rate uses a negative feedback technique. The correction formula contains compensation elements of actual modification and trends in glucose concentration (full equation set for euglycemic clamp in [5]). This algorithm was tested within a predictive window of our experimental working range, ranging from 5 to 25 ml/min. Figure 8 shows the results of a regression analysis between the feedback control algorithm output and the target experimental rates, as calculated in Period-II. A correlation coefficient of 0.9265 was obtained between the predicted and the desired rates. The average calculated error in glucose infusion rate was 9.2±1.1%. It can also be seen that the feedback control algorithm performs better in the middle range of infusion rates, therefore suitable for more particular protocols and subjects. Our ANN control algorithm shows better results while using a low blood sampling rate and a wide range of conditions, make it applicable for both animal and human studies.

## Discussion

Artificial neural networks models have been effectively used in various health care applications, for example, classification of heart rate variability for clinical diagnosis such as arrhythmia [18], [19] and ischemia [20], diagnosis based on gene expression signature [21] and image pattern recognition associated with cancer [22]. Due to its multidimensional nature, ANN has also been used for diagnosis of diabetes based on physical parameters [23], [24], prediction of blood glucose levels [25], [26], [27], regulation of insulin dosage setting algorithms for insulin pumps [28] and detection of hypoglycemic episodes in type I diabetes patients [29]. We proposed the use of artificial neural networks for automatic regulation of glucose infusion rate during HEGC study. Automatic real-time adjustment of glucose infusion rate during glucose clamp test was examined in several studies for its potential to increase experimental reliability and validity and to ease the examination procedures. Our results support this understanding and demonstrate that artificial neural networks method is a powerful tool for real time control of glucose levels during HEGC. As described in the results, the prediction ability of the proposed ANN controller during the experimental phase of interest was found to be more accurate than the feedback control that was proposed by DeFronzo *et al.* when applied at equivalent low sampling interval (prediction error of 3.7% vs. 9.2% respectively. Other control feedback algorithms have provided similar performance measures [5], [6], [7], [8], [30]). Recently, predictive control ANN based strategy was used for solving similar problems, set-point tracking, and system output maintenance [31]. This strategy is designed for high sampling rate or long lasting procedures and was not considered here due to the preferable low sampling rate of blood.

Training a specific neural network with different initial conditions may yield different sets of connection weight, and therefore different performance rates. Since our network was trained with random initial conditions (initial weights), the system’s generalization performance was determined based on ensemble ANN technique, where multiple networks are created through different conditions. The desired output prediction of the glucose pump controller was determined base on the ensemble performance instead of a single network prediction. This output may be less varied and contribute to the system’s stabilization. It has been shown that the use of an ANN ensemble that differs only in its initial conditions (but with same architecture and training sets) may reduce significantly the variance in prediction error [32]. A common type of determination of ensemble performance is the ensemble averaging. In this method the output prediction is the average of the ensemble networks output. We chose to use the median value of an ensemble of 50 networks trained separately, since using a median value may reduce the effect of networks with poor training performance, and avoid the contribution of extreme values [33]. Based on Period-II results, we can conclude that artificial neural networks based control strategy is a powerful tool to control plasma glucose level, taking into account its stochastic nature. We assume that larger database of Period-I may enhance generalization of the network and improve performance rates.

Genetic algorithms (GA) are usually used for solving robust optimization problems and were previously used to optimize network’s weights [34], [35], to optimize neural networks ensemble mainly in classification problems [36], or to realize novel architectures in the form of connectivity patterns [37], [38]. In our study an accurate value of glucose infusion rate was needed. We have, therefore, used GA in order to optimize network topology and learning features, instead of using trial and error approach which is a common approach for ANN topology determination. In our case it was preferable especially for evaluating the learning parameters.

A possible drawback of feedback control algorithms and ANN based control solutions is the specificity of the experimental protocol. Feedback control algorithms include empirical parameters suitable to very particular protocols and subjects. Our experiments were preformed with similar protocol: specific primed continuous dosage of insulin, infusion rates, fasted animals and examination at the same time of the day. On the other hand, our predictive window covers a range of experiments with different rat species (Wistar, Sprague Dawley, Fischer-F344 rats), body weights and types of treatment that might be used in insulin resistance research.

Finally, the system was trained with data obtained from manually controlled experiments, where variations in plasma glucose level are sometimes larger then desired (our training data set included variations in plasma glucose of up to 20%). When using data of clamp experiments that are manually set (instead of data obtained from clamp control by automatic systems) experimental errors may include: blood measurement, pump rate, operator, etc. These experimental errors may be interpreted as random noise, such that using manually set data allows a variety of dynamic events and situations that increase the controller’s prediction power. Furthermore, good prediction power was obtained under different levels of noise introduced with the input vectors. Thus we conclude that the precision and robustness of the model may reflect its stability in real time cases where blood glucose should be kept within the predictive window of the controller.

To summarize, assessment of insulin sensitivity is highly valuable in glucose homeostasis research, in the development of drugs for diabetes and in clinical diagnostics. The results presented here, offer a reproducible and most accurate method for regulation of glucose levels during hyperinsulinemic-euglycemic glucose clamp test. Such real-time automatic system may expand the use of this technique to a boarder range of applications and make it feasible to use in large scale studies.
